# *QuickStats:* Percentage of Adults Aged ≥18 Years with Any Hearing Loss,* by State — National Health Interview Survey,^^† ^^2014–2016

**DOI:** 10.15585/mmwr.mm6650a7

**Published:** 2017-12-22

**Figure:**
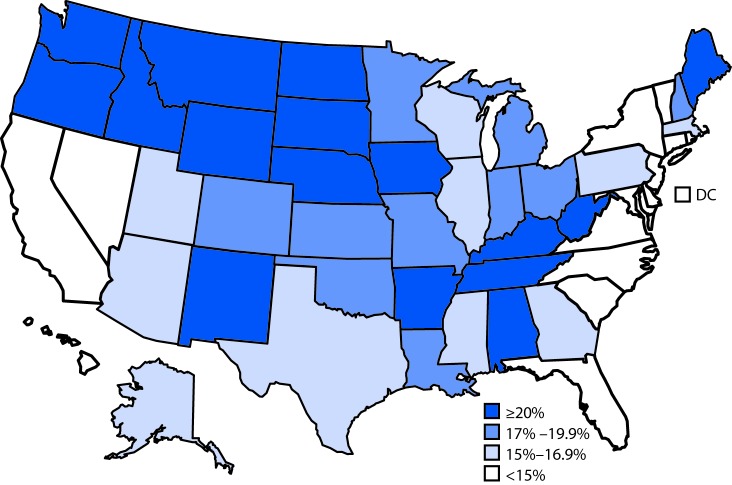
Overall, 15.9% of U.S. adults aged ≥18 years had any hearing loss during 2014–2016. The prevalence of any hearing loss was lowest in New Jersey (10.6%), Connecticut (11.0%), Maryland (11.0%), California (12.3%), New York (12.6%), and the District of Columbia (8.6%). The prevalence of any hearing loss was highest in West Virginia (24.7%), Oregon (24.6%), Montana (23.8%), Idaho (23.1%), and Wyoming (22.3%).

**Source: **National Health Interview Survey, 2014-2016. Survey data available at https://www.cdc.gov/nchs/nhis/data-questionnaires-documentation.htm. Tabular results available at https://www.cdc.gov/nchs/data/health_policy/hearing_loss_table_SEs.pdf.

